# Hydrogel-Based Delivery Systems for Targeted Probiotic Release and Colonization: From Design Principles to Health Outcomes

**DOI:** 10.3390/gels12100914

**Published:** 2026-10-10

**Authors:** Wenjia Yan, Haichuan Shi, Hao Wang, Shanshan Xu, Zhuochen Wang

**Affiliations:** 1 Institute of Agro-Products Processing, Anhui Academy of Agricultural Sciences, Hefei 230001, China; sevenyan@cau.edu.cn (W.Y.); whdamonxin@163.com (H.W.); xss8894@163.com (S.X.); 2 Anhui Engineering Laboratory of Food Microbial Fermentation and Functional Application, Hefei 230001, China; 3 School of Food and Nutrition, Anhui Agricultural University, Hefei 230036, China; 17581848629@163.com

**Keywords:** hydrogels, probiotic delivery, oral administration, targeted release, gut colonization

## Abstract

Probiotics exert profound health benefits via the modulation of the intestinal microbiota. However, the efficacy of orally administered probiotics is severely constrained by a cascade of physiological barriers, including gastric acid inactivation, suboptimal intestinal release, and inadequate mucosal colonization. In response to these challenges, hydrogel-based delivery platforms have garnered considerable interest as versatile carriers capable of providing protective encapsulation, enabling spatiotemporally controlled release, and augmenting intestinal retention. This narrative review presents recent progress in the design and application of hydrogel-based oral delivery systems tailored for targeted probiotic release and effective colonization. First, the key gastrointestinal obstacles to successful probiotic delivery are delineated. Hydrogel design parameters are then critically appraised, with particular emphasis on material selection and network design. The underlying release mechanisms are subsequently examined. These encompass pH-responsive, enzyme-triggered, and inflammation-responsive modalities. Emerging colonization-enhancing strategies are discussed in detail. These include mucoadhesive hydrogel architectures and the nascent paradigm of “living hydrogels” functioning as dynamic bioreactors. The translational potential of these systems for health management is illustrated through applications in inflammatory bowel disease, colorectal cancer, and gut–brain axis modulation. Finally, key challenges in manufacturing, viability, and translational feasibility are evaluated. These include regulatory, safety, and encapsulation-related constraints. To address these, multi-responsive platforms, machine learning-assisted design, and synbiotic hydrogels are proposed. These hydrogels synergize with probiotics to target dysbiosis and repair mucosal damage for improved gastrointestinal health management.

## 1. Introduction

The definition of probiotics was first proposed by the World Health Organization (WHO) and the Food and Agriculture Organization (FAO) in 2001. It was revised in 2014 as “live microorganisms that, when administered in adequate amounts, confer a health benefit on the host” [1]. Most probiotics are classified as Lactobacillus, Bifidobacterium, or Saccharomyces (yeast) [2]. Probiotics have been reported to confer multiple health benefits, including the prevention of colorectal cancer [3], the prevention of inflammatory bowel disease [4], the prevention of irritable bowel syndrome [5], a reduction in cardiovascular disease risk [6], cholesterol-lowering and lipid-lowering effects [7], and the prevention of non-alcoholic fatty liver disease-associated hepatocellular carcinoma [8].

It has been established that probiotic strains must reach the intestinal tract at a viable count of at least 10^6^ CFU·g^−1^ to exert beneficial effects on human health [9]. However, viability is substantially reduced during gastrointestinal transit, which presents two main barriers. The first is gastric acid and bile salts, which disrupt membrane potential and permeability, leading to cell death [10]. The second is the resident gut microbiota, which inhibits colonization by exogenous strains and limits the efficacy of even acid-tolerant probiotics [11,12]. To overcome these barriers, advanced delivery systems are needed to enhance survival, enable targeted release, and promote site-specific colonization, thus allowing probiotics to perform their physiological functions. Hydrogel-based delivery systems have garnered attention as effective probiotic carriers. They form stable matrices that shield probiotics from hostile environmental conditions [13]. They also exhibit intelligent responsiveness to external stimuli. This responsiveness enables the controlled release of the encapsulated probiotics [14].

Evaluating delivery systems requires distinguishing the sequential stages of the probiotic journey [15,16]. Encapsulation protects bacteria during fabrication. Gastrointestinal survival maintains viability under harsh host stressors. Controlled release determines site-specific liberation from the carrier. Mucosal adhesion initiates cellular binding to the intestinal epithelium. Mucosal retention measures the duration of localized presence before clearance. Colonization is not equivalent to transient retention; rather, it requires successful niche competition, sustained replication, and long-term population persistence. Therapeutic effects are downstream physiological benefits driven by stable engraftment. These stages provide a framework for assessing hydrogel carriers, connecting design principles to targeted release, colonization, and health outcomes.

To identify relevant literature, a search was conducted in Web of Science, Scopus, PubMed, and Google Scholar for studies published between 2000 and 2026. The main search terms were “probiotic”, “gel”, “encapsulation”, “delivery system”, “viability”, “gastrointestinal transit”, “controlled release”, and “colonization”. Studies were considered relevant if they addressed probiotic encapsulation, hydrogel/gel-based delivery systems, viability, gastrointestinal transit, controlled release, or colonization. Conference abstracts, editorials, and letters were excluded. As this is a narrative review, no formal systematic screening or risk-of-bias assessment was performed. Based on this search, this review explores the role of hydrogel-based delivery systems in improving probiotic survival. It evaluates their effectiveness in food applications and highlights major challenges and future research directions.

## 2. Gel Matrix Design and Fabrication Strategies

### 2.1. Hydrogel

#### 2.1.1. Hydrogel Design Strategies by Biopolymer Type

Based on ingredient categories, hydrogels are categorized into three types: polysaccharide, protein, and starch. Polysaccharide-based hydrogels have emerged as promising carriers for probiotic delivery. This is due to their inherent biocompatibility, biodegradability, and abundant availability [17]. Common polysaccharides used in probiotic encapsulation include alginate, hyaluronic acid, chitosan, carrageenan, pectin, cellulose, nanocellulose, starch, gellan gum and xanthan gum. The macromolecular architecture of polysaccharide gels provides structural stability that helps shield probiotics from harsh processing and gastric conditions [18]. Non-starch polysaccharides can withstand the host upper GIT environment. This environment includes gastric acid, bile salts, degradation by mammalian enzymes, and gastrointestinal absorption. They can also be fermented by gut biota. The polysaccharide backbone possesses abundant functional groups, including carboxyl, amino, hydroxyl, and aldehyde moieties. The resulting strong affinity of polysaccharide gels for the intestinal mucosal layer facilitates probiotic residence and colonization [19,20]. Therefore, such polysaccharide hydrogels have been applied as colon-targeted delivery systems for probiotics. Despite these natural advantages, hydrogels composed of a single polysaccharide species often exhibit inherent limitations. These include poor mechanical strength, larger network pores, low encapsulation efficiency and insufficient structural integrity. To overcome these shortcomings, researchers have turned to blending, chemical modification, or the incorporation of reinforcing agents. Polysaccharide–polysaccharide or polysaccharide–protein composite hydrogels have been fabricated to improve probiotic viability and delivery performance. Alginate can form complexes with chitosan [21], carboxymethyl chitosan [22], κ-carrageenan [23], pectin [24,25,26,27,28,29], and starch [30] via noncovalent interactions, such as electrostatic interactions, hydrophobic interactions and hydrogen bonding. The resulting complex coacervates create a reinforced gel matrix that offers superior protection against acidic environments and bile salt. Mehta et al. (2025) [31] found that the gellan–arabinoxylan hydrogel combines structural stability and prebiotic activity. It supports targeted probiotic delivery and gut health promotion. In addition to polysaccharide–polysaccharide composite hydrogels, protein–polysaccharide hydrogels are also widely utilized. The incorporation of protein imparts mechanical toughness to withstand peristaltic shear, while its high water-holding capacity retards gastric acid ingress and complements the buffering effect of ionizable protein residues. The co-assembled network features a denser and more uniform pore structure. This structure physically excludes pepsin and bile salts. It confines probiotics until intestinal protease degradation. It also permits nutrient diffusion. Enzymatic hydrolysis of the protein backbone then enables targeted small-intestinal release. It liberates peptides and amino acids. These serve as nitrogenous substrates for probiotic resuscitation and colonization. This is a nutritional synergy beyond the reach of polysaccharide-only matrices. These integrated properties render protein–polysaccharide hydrogels a resilient and physiologically attuned delivery vehicle, independent of the specific crosslinking strategy [32,33]. For example, whey protein isolate has been combined with Momordica charantia polysaccharide via noncovalent interactions. It has also been conjugated with soybean hull polysaccharide through the Maillard reaction. Both provided superior protection to *Lactobacillus acidophilus* during freeze-drying and in vitro digestion compared to WPI alone [34,35]. Other systems, such as gelatin–polysaccharide and soy protein isolate–pectin composites [36], similarly exploit the structural and nutritional advantages of the protein component. This enhances probiotic delivery. Specific prebiotics have been integrated into symbiotic probiotic hydrogels. These notably include oligosaccharides and polyphenols. Oligosaccharides promote diverse beneficial gut microbes including *Akkermansia muciniphila* and *Faecalibacterium prausnitzii*. They inhibit opportunistic pathogens like *Clostridioides difficile*. They also fortify the intestinal barrier. They modulate immune or metabolic signaling [37,38,39]. Pectic oligosaccharides introduced into pea protein microgel-reinforced low-methoxyl pectin hydrogel beads created a denser network, which enhanced encapsulation efficiency and colonic probiotic release [40]. Polyphenols, aside from their antimicrobial properties, serve as prebiotic substrates through both intact structures and metabolites. In this way, they reshape the gut microbiota [41]. Yu et al. (2026) [42] found metal–phenolic networks reinforced sodium caseinate–gellan gum hydrogels, markedly improving antioxidant capacity and encapsulation efficiency. Zhu et al. (2024) [43] demonstrated that tannic acid plays a dual role as both an antioxidant and a prebiotic nutrient. Through hydrogen bonding and hydrophobic associations, tannic acid densified the hydrogel matrix. This enhanced bacterial survival during simulated digestion and refrigerated storage.

#### 2.1.2. Hydrogel Design Based on Network Structure

Based on their network structure, hydrogels for probiotic delivery can be broadly classified into single-network hydrogels, semi-interpenetrating network (semi-IPN) hydrogels, and interpenetrating network (IPN) hydrogels (Table 1). Single-network hydrogels are the simplest to prepare using a single type of molecule. Alginate has been crosslinked using calcium ions to achieve the delivery of probiotics [44]. Wang et al. (2025) [45] revealed that Ulva lactuca polysaccharides can form a heat-resistant gel through Fe^3+^ cross-linking involving hydroxyl and carboxyl groups, and the resulting gel microspheres effectively protect probiotics against low pH and bile salt-induced damage.

Semi-interpenetrating polymer network (semi-IPN) hydrogels are formed by introducing linear or branched macromolecules into a crosslinked polymer network. The non-crosslinked chains penetrate the network at the molecular level without destroying its overall structure. This architecture can adjust network density, porosity, and swelling behavior. It also preserves the integrity of the gel matrix. Sodium alginate is one of the most widely studied polysaccharides for probiotic delivery. This is due to its biocompatibility, mild gelation conditions, and ease of processing. It is obtained either from bacterial fermentation or from marine brown algae. It is composed of 1,4-linked β-D-mannuronic acid (M) and α-L-guluronic acid (G) residues. These residues are arranged in homopolymeric M-blocks, G-blocks, and alternating MG sequences. In aqueous solution, alginate readily forms hydrogels through ionic crosslinking between its carboxyl groups and divalent cations. This gelation behavior depends strongly on the M/G ratio, with G-blocks playing the dominant role in forming the characteristic “egg-box” structure. Building on these advantages, recent studies have incorporated a second polysaccharide (e.g., carrageenan, gum arabic, κ-carrageenan, low-methoxyl pectin, and konjac glucomannan [46], and low-acyl gellan gum [47]) into alginate-based systems to construct denser semi-IPNs with improved mechanical stability and barrier properties. Zhu et al. (2019) [48] developed a novel semi-interpenetrating network hydrogel by cross-linking peach gum polysaccharide and Auricularia polytrichid β-glucans via COO^−^–Fe^3+^ coordination. They found that the hydrogel effectively protected probiotics during gastrointestinal transit and enabled controlled release under simulated intestinal conditions.

Interpenetrating polymer network (IPN) hydrogels consist of two or more networks that are interlaced at the molecular scale but not covalently bonded to each other. This unique architecture endows IPN gels with several features. These include a dense, homogeneous microstructure, numerous chain entanglements, and synergistic physicochemical properties. The formation of IPN gels typically employs either sequential or simultaneous crosslinking strategies. These strategies are triggered by physical factors (e.g., heating–cooling, pH, UV irradiation), chemical factors (e.g., metal ions or organic crosslinkers like genipin or glutaraldehyde), or enzymatic factors (e.g., transglutaminase, laccase, thrombin, and horseradish peroxidase). Notable examples include alginate–gelatin IPNs crosslinked by Ca^2+^ and transglutaminase, chitosan–gelatin networks crosslinked by genipin, and fibrin–silk fibroin systems formed via dual-enzyme catalysis. CMC-κ-car IPNs showed a higher EE (94.7%) than CMC gel (74.2%) for the encapsulation of probiotic bacteria. These IPNs can also be applied to release bioactive compounds in a controlled manner [49]. Wang et al. (2026) [50] constructed a Hericium erinaceus polysaccharide–chitosan oligosaccharide composite hydrogel with an IPN structure. This hydrogel overcomes polysaccharide mechanical and gastric instability. It significantly enhances probiotic survival and targeted intestinal release. It thereby provides a novel biomaterial and rheological design framework for probiotic delivery. An emulsion-templated IPN microgel system was developed. It is based on pea protein isolate and low-methoxyl pectin. It is developed for targeted intestinal delivery of a uric-acid-degrading probiotic. It demonstrated exceptional protective performance during in vitro digestion and preservation of metabolic bioactivity. This highlights its potential as an effective oral probiotic carrier [51]. Compared to single-network or semi-IPN gels, IPN gels exhibit significantly enhanced mechanical strength, thermal stability, freeze–thaw resistance, water retention, and swelling controllability, while retaining the inherent biocompatibility and biodegradability of their natural biopolymer constituents (e.g., polysaccharides and proteins). However, a critical trade-off exists among these architectures: while advanced IPN systems provide superior mechanical protection and sustained release profiles during harsh gastric transit, their complex cross-linking chemistry and multi-step fabrication processes significantly increase formulation complexity and limit industrial scalability. In contrast, single-network and semi-IPN hydrogels, despite their lower mechanical resilience, offer higher reproducibility and ease of manufacturing, making them more attractive for cost-effective, large-scale probiotic food applications.

**Table 1:** Hydrogel systems embedded with probiotic.

Hydrogel Structure	Composition	Crosslinking Method	Probiotic Strain	Encapsulation Efficiency (%)	Experimental Model	Digestion Survival	Storage Temp./Time	Storage Survival	Ref.
Single network	Alginate	Crosslinked by Ca^2+^	*Lactobacillus casei* ATCC 393	Not mentioned	In vitro	6.88 log CFU/g	4 °C for 28 days	7.13 log cfu g^−1^	[44]
	Ulva lactuca polysaccharides	crosslinked by Fe^3+^	*Lactobacillus gasseri*	50 ± 3.0%	In vitro	13.0%	Not mentioned	Not mentioned	[45]
Semi-IPN	Sodium alginate and gum arabic (A-A), κ-carrageenan (C-A), low-methoxyl pectin (P-A), konjac glucomannan (K-A), low-acyl gellan gum (G-A), and xanthan gum (X-A)	Crosslinked by Ca^2+^	*Limosilactobacillus fermentum*	80–100%	In vitro	SIF: 7.98–8.07 log CFU/g.	Not mentioned	Not mentioned	[46]
	Sodium alginate and low-acyl gellan gum	Crosslinked by Ca^2+^	*Lactobacillus helveticus* and *Lactobacillus delbrueckii* spp. *lactis*	10^6^–10^7^ CFU/g, 4.56–92.83%	Not mentioned	Not mentioned	Not mentioned	Not mentioned	[47]
	Peach gum polysaccharide and auricularia polytrichid β-glucans	Crosslinked by Fe^3+^	*Lactobacillus salivarius* CICC23174 and *Lactobacillus plantarum* CICC20264	91.98 ± 0.035% and 89.22 ± 0.097%	In vitro	stomach release:0.602 ± 0.23 (*L. salivarius*) and 0.204 ± 0.36 (*L. plantarum*) log CFU/g, duodenum > 1.5 log CFU/g (trypsin/bile salts); caecum > 7 log10 CFU/g. Overall loss 1–2 log10 CFU/g, cells still viable	Not mentioned	Not mentioned	[48]
IPN	carboxymethyl cellulose and k-carrageenan	Crosslinked by Ca^2+^ and K^+^	*Lactobacillus plantarum* ATCC:13643	86.5–94.7%	In vitro	6.87 Log CFU/g	Storage at 4 °C for 1 month	No significant viability loss	[49]
	Hericium erinaceus polysaccharide–chitosan oligosaccharide	70 °C for 30 min, then allowed to cool naturally to room temperature	*Lactobacillus plantarum* (LP) and *Escherichia coli* Nissle 1917 (EcN)	Not mentioned	In vitro	LP 79–87%, EcN 68–72%	Not mentioned	Not mentioned	[50]
	Pea protein isolate and low-methoxyl pectin	GDL and Ca^2+^	*Lacticaseibacillus paracasei* YWC-01	42.4–99.5%	In vitro	>9 log_10_ CFU/g with <0.4 log reduction over 4 h	Not mentioned	Not mentioned	[52]
	Sugar beet pectin (SBP) and soybean protein isolate (SPI)	Laccase	*Lactobacillus paracasei* LS14	>88.9%	In vitro	66.37–6.93 Log of CFU/ml	Storage at 4 °C for 21 days	No significant change for 10% SPI/3.5% SBP/10 U	[53]

Note: Experimental models are categorized as In vitro (simulated digestion) and Animal (in vivo animal models, with species specified). “Not mentioned” indicates that the experimental model was not clearly reported in the original study. Release behavior and main outcomes were not added as separate columns because the included studies differed substantially in release media, pH, time points, analytical methods, and outcome definitions, making consistent comparison inappropriate. CFU, colony-forming units.

**Table 2:** Multiphase gel systems embedded with probiotic.

Structural Category	Composition	Preparation Method	Probiotic Strain	Encapsulation Efficiency (%)	Experimental Model	Digestion Survival	Storage Temp./Time	Storage Survival	Ref.
Emulsion gel	Whey protein isolate, high-acyl gellan gum	85 °C for 30 min	*Lactobacillus plantarum* LP90	80.43–94.10%	In vitro	7.20–7.72 log CFU/g	4 ℃/3 months	6.96–7.53 log CFU/g	[54]
	Wheat germ oil, whey protein isolate,	sonication	*Lacticaseibacillus rhamnosus* B-442	~97%	Not mentioned	Not mentioned	4 ℃/21 days	7.78 log CFU/mL	[55]
	C-phycocyanin–pectin	CaCl_2_	*Lactobacillus plantarum*	Not mentioned	In vitro	7.14 log CFU/g	4 ℃/35 days	8.98 log/CFU	[56]
	Xanthan gum, tea protein	45 °C for 4 h.	*Bifidobacterium lactis*	Not mentioned	In vitro	5.92 log CFU/g	4 °C/11 days	Above 8.00 log CFU	[57]
	Soy hull polysaccharide, soybean protein isolate	GDL	*Lactobacillus plantarum*	99.0%	In vitro	8.37 × 10^9^ CFU/mL	Not mentioned	Not mentioned	[58]
					Animal (Zebrafishs)	1.18 × 10^7^ CFU/mL after 14 days of feeding			
W_1_/O/W_2_ double emulsion gel	Soybean saponin, soy protein isolate, sodium alginate	CaCl_2_	*Lactobacillus plantarum*	>99%	In vitro.	91.96%	Not mentioned	Not mentioned	[59]
	sodium caseinate–κ Carrageenan by Maillard reaction, sodium alginate–carboxymethyl chitosan gel	Citric acid	*Lactobacillus rhamnosus* 76	Not mentioned	In vitro	90.69%	Not mentioned	Not mentioned	[60]
					Animal (C57BL6/J mice)	88.08%			
	Soybean oil, gelatin	TGase	*Lactobacillus rhamnosus* 76	Not mentioned	In vitro	Not mentioned	25 °C/4 weeks	>10^6^ CFU/g	[61]
	Rapeseed, whey protein isolate	Carnauba was organogelator, WPI was gelled via CaCl_2_ treatment	*Lactobacillus plantarum*	Not mentioned	Not mentioned	Not mentioned	4 °C/56 days	6.69–7.78 log CFU/g	[62]
Bigel	Soybean oil, flaxseed gum	beeswax and soybean lecithin act as organogelators	*Lactobacillus plantarum*	64.26–90.75%	In vitro	6.80–7.33 log CFU/g	4 °C/6 weeks	6.21–6.59 log CFU/g	[63]
	Sesame oleogel and alginate hydrogel	Beeswax act as an organogelator, alginate was gelled via ion interaction	*Lacticaseibacillus casei* NRRL B-1922 and *Lactiplantibacillus paraplantarum* NRC AM	Not mentioned	Not mentioned	Not mentioned	4 °C/120 days	7.45 and 7.83 log CFU/g	[64]
	soybean oil, soy protein isolate, soy hull polysaccharide	Soy lecithin and stearic acid as oleogelators, hydrogel formed ny heating at 85 °C for 30 min	*Lactobacillus plantarum*	90.3%	In vitro	>60%	4 °C/4 weeks	5.47 × 10^9^ CFU/g	[52]

Note: Experimental models are categorized as In vitro (simulated digestion) and Animal (in vivo animal models, with species specified). “Not mentioned” indicates that the experimental model was not clearly reported in the original study. Release behavior and main outcomes were not added as separate columns because the included studies differed substantially in release media, pH, time points, analytical methods, and outcome definitions, making consistent comparison inappropriate. CFU, colony-forming units.

### 2.2. Emulsion Gel

As an emerging structural platform, emulsion gels provide a robust spatial matrix designed to safeguard sensitive bioactives against environmental degradation. The emulsion gel, a solid or semi-solid colloidal dispersion, has emerged as a highly promising delivery platform due to its distinctive three-dimensional network architecture. Compared to single emulsions or gels, hybrid emulsion–gel systems exhibit superior physical stability and tunable properties through versatile structural design [65]. The dense matrix physically entraps probiotics, creating a protective barrier against heat, oxygen, gastric acid, and digestive enzymes [66] (Table 2).

Beyond simple network formation, tailoring biopolymer composition serves as a primary strategy to enhance matrix density and environmental resistance. This tailoring is particularly achieved through protein and polysaccharide combinations. Among various biopolymers, starch-based emulsion gels offer high biocompatibility and ease of modification. Notably, an elevated amylose content slightly weakens gel integrity. However, it markedly improves probiotic survival during thermal processing and simulated gastrointestinal (GI) digestion [67]. Protein–polysaccharide composite systems similarly optimize matrix density and viscoelasticity. For instance, tuning ovalbumin concentration with transglutaminase crosslinking enhances the post-digestion survival of *Bifidobacterium lactis* [68], while milk fat globule membrane (MFGM)–pectin [69] and whey protein isolate (WPI)–gellan gum matrices [54] significantly improve storage stability and thermal protection for *Lactobacillus plantarum* by reducing water activity and reinforcing network strength (Table 2).

Complementing structural polymers, the integration of bioactive secondary functional ingredients into the gel matrix provides supplemental antioxidant and protective benefits. Incorporating bioactive compounds into these matrices provides additional protective synergy. C-phycocyanin–pectin beads co-encapsulating procyanidin [56] and gelatin–sodium alginate hydrogel beads modified with tannic acid [70] both utilize dense microstructures and abundant hydroxyl interactions to enhance antioxidant capacity and cell survival under GI stress. Similarly, sonicated emulsion gels enriched with orange essential oil preserve *Lactobacillus* viability up to 97.66% in yogourt while retarding lipid oxidation [55].

To overcome severe processing and physiological barriers, researchers have developed advanced interfacial engineering techniques. These techniques include Pickering stabilization and high-internal-phase systems. They are designed to achieve superior stability and targeted release. Tea protein/xanthan gum Pickering emulsion gels exhibit tunable rheology suitable for 3D printing, maintaining *L. plantarum* viability up to 65 °C and during chilled storage [57]. In high-internal-phase emulsion gels (HIPEs), increasing gluconolactone crosslinks soy protein particles to promote β-sheet structures, achieving up to 99.0% encapsulation efficiency and enhanced intestinal colonization [55,58]. Furthermore, water-in-oil jammed Pickering emulsion gels (JPEGs) shield probiotics from gastric acidity while enabling lipase-triggered, site-specific release in the intestine [71].

### 2.3. W_1_/O/W_2_ Double Emulsion Gel

To overcome these thermodynamic instabilities, researchers immobilize double emulsions within three-dimensional gel networks or hydrogel bead shells. These matrices suppress droplet migration, attenuate oxygen diffusion, and confer robust mechanical protection [59,60,61,72]. Recent studies demonstrate that such gel-stabilized double emulsion systems significantly enhance probiotic survival and delivery efficacy under hostile conditions (Table 2). For instance, structuring the oil phase with carnauba wax or crosslinking polymeric shells preserves cell viability across prolonged storage and freeze–thaw cycles [61,62]. Enzymatic- or Maillard-reaction-assisted crosslinking optimizes gastrointestinal tolerance and enables targeted intestinal release [59,60,61]. Furthermore, these tailorable architectures facilitate complex co-delivery strategies, successfully synchronizing probiotics with prebiotics, therapeutic agents, or engineered microbes to modulate gut microflora and enhance metabolic outputs in intestinal models [72].

### 2.4. Bigel

Bigels, formed by homogenizing an oleogel and a hydrogel phase under controlled shear and temperature, represent an innovative dual-phase delivery platform capable of co-encapsulating lipophilic and hydrophilic active agents (Table 2). By merging the structural networks of both gels, bigels exhibit superior viscoelasticity, thermal stability, and physical robustness compared to conventional single emulsions. Critically, this composite architecture overcomes the inherent limitations of single-phase systems. It mitigates the rapid diffusion typical of hydrogels. It also alleviates the restricted release of water-soluble compounds from oleogels. Consequently, it provides an adaptable matrix for controlled bioactive delivery. When applied to probiotic preservation, bigels leverage their dense, compartmentalized microstructures to confer exceptional physical protection against acidity, thermal processing, and environmental degradation. Formulations utilizing soy-derived polymers, whey proteins, beeswax, or plant gums regularly achieve encapsulation efficiencies exceeding 90% for strains such as *Lactiplantibacillus plantarum*, *Lactobacillus acidophilus*, and *Bifidobacterium lactis* [63,64,73]. These systems significantly extend storage viability over prolonged periods (up to six weeks) in acidic food matrices like yogurt and dramatically improve survival under simulated gastrointestinal conditions. However, phase behavior remains a critical design parameter; while optimal oleogel-to-hydrogel ratios (typically around 5:5) maximize structural integrity and cell protection, excessive oil fractions can induce phase inversion that compromises probiotic viability, underscoring the necessity of precise phase tuning for bigel-based functional foods [52].

Among advanced multi-phase delivery systems, emulsion gels, double-emulsion gels, and bigels exhibit distinct trade-offs in structural complexity, protection efficacy, and industrial scalability. Emulsion gels, featuring oil droplets embedded within a continuous biopolymer network, offer a balanced approach with moderate probiotic protection and high manufacturing feasibility. However, for harsh gastrointestinal conditions or oxygen-sensitive strains, double-emulsion gels (W_1_/O/W_2_) provide superior multi-barrier protection through their core–shell architecture, though at the cost of high formulation complexity and susceptibility to phase destabilization during scaling up. Conversely, bigels structured by interpenetrated hydrogel–oleogel networks exhibit exceptional utility in co-delivering probiotics and lipophilic payloads, supported by superior barrier properties against moisture and oxygen. Despite their excellent probiotic loading capacities, bigels and double-emulsion gels currently face limitations in industrial-scale production compared to conventional emulsion gels, necessitating future research into formulation optimization and cost-effective manufacturing.

## 3. Mechanisms of Targeted Release and Enhanced Gut Colonization

**Figure 1:**
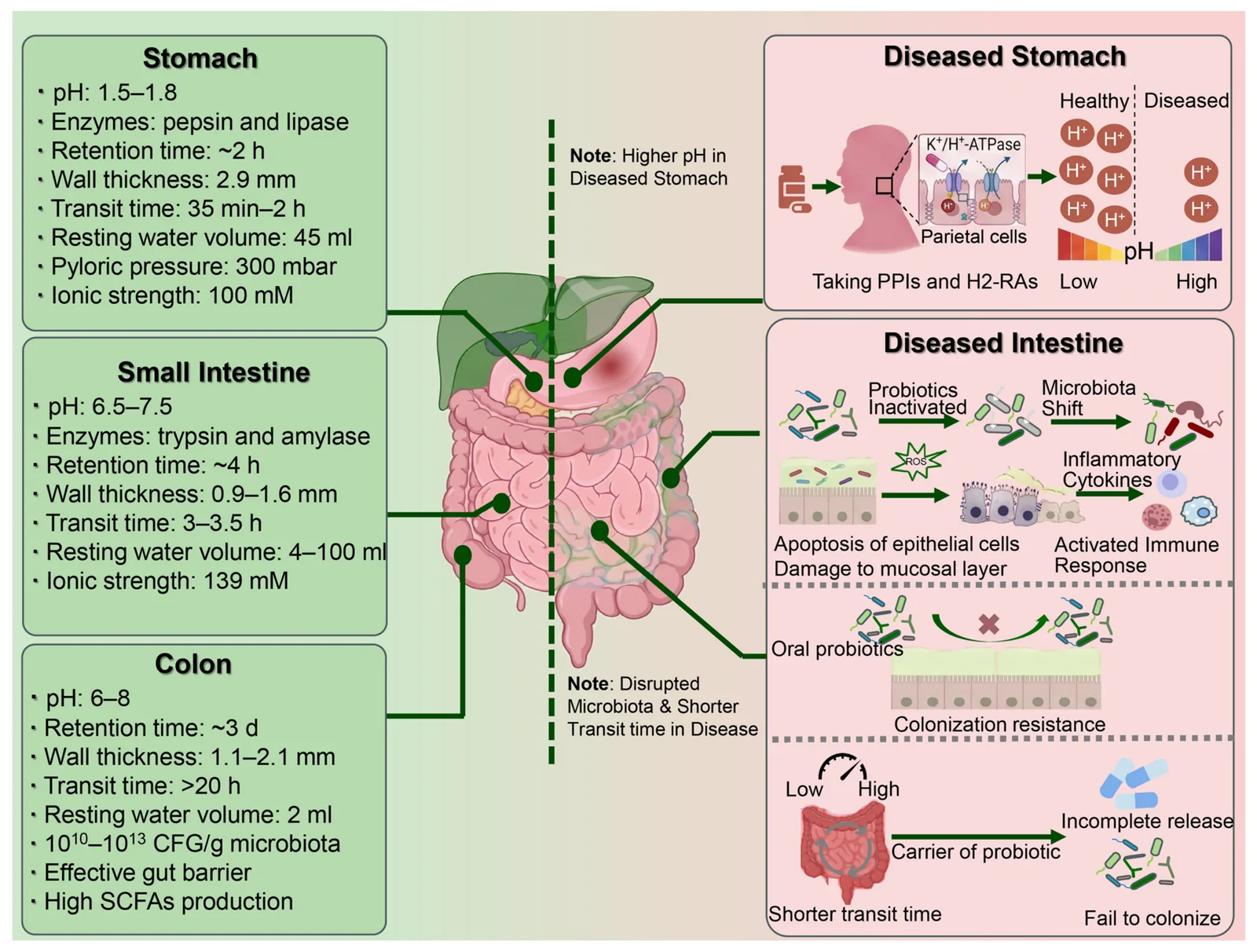
Gastrointestinal physiological environment under normal and pathological conditions.

Achieving effective oral delivery of probiotics necessitates overcoming the highly dynamic and formidable barriers of the gastrointestinal (GI) tract. Meeting this requirement is critical for both preserving healthy homeostasis and treating pathological conditions. In a normal physiological state, unprotected probiotics must survive a brief oral transit before navigating a harsh sequence of environments, including extreme gastric acidity, fluctuating enzymatic profiles, and varying transit times across the small and large intestines (Figure 1). Consequently, delivery systems must be rationally designed to dynamically interpret these sequential physicochemical signals to achieve site-specific and kinetically controlled release. This baseline delivery challenge is severely compounded during gastrointestinal disease, where the physiological landscape undergoes drastic alterations. Medical interventions, such as the use of proton pump inhibitors (PPIs), elevate gastric pH, while the intestinal environment frequently suffers from microbiota dysbiosis, accelerated transit times, and mucosal barrier degradation. These pathological shifts culminate in a profound loss of colonization resistance, causing therapeutic agents to be prematurely inactivated or rapidly expelled. To counteract these cumulative barriers across both healthy and diseased states, advanced gel-based delivery networks are engineered to actively negotiate the gut’s complex chemical and mechanical landscape. The following sections systematically examine the mechanisms by which these polymeric matrices achieve successful transit and robust gut colonization. We first explore how stimuli-responsive targeted release designs utilize local environmental triggers to dictate precise, site-specific liberation. This is followed by an analysis of mucoadhesive and mucus-penetrating designs, which are specifically tailored to counteract rapid transit and prolong therapeutic residence at the epithelial interface.

### 3.1. Stimuli-Responsive Targeted Release

#### 3.1.1. pH-Responsive

The deployment of stimuli-responsive architectures, specifically pH-sensitive hydrogels, has become a cornerstone strategy for the oral delivery of living therapeutics. By exploiting the distinct physiological pH gradients traversing the gastrointestinal (GI) tract, these smart platforms prevent premature bacterial degradation and ensure site-specific colonization [74]. Architecturally, these systems operate on two distinct spectra: low-pH-responsive formulations engineered for highly acidic microenvironments, such as tumors [75,76], and high-pH-responsive frameworks optimized for GI transit, which shield against corrosive gastric acids before triggering release in the neutral or weakly alkaline intestine [77]. To achieve this precision, carriers rely on dynamic physicochemical shifts encompassing two overarching release paradigms: degradation-induced liberation and expansion-driven swelling [78].

**Figure 2:**
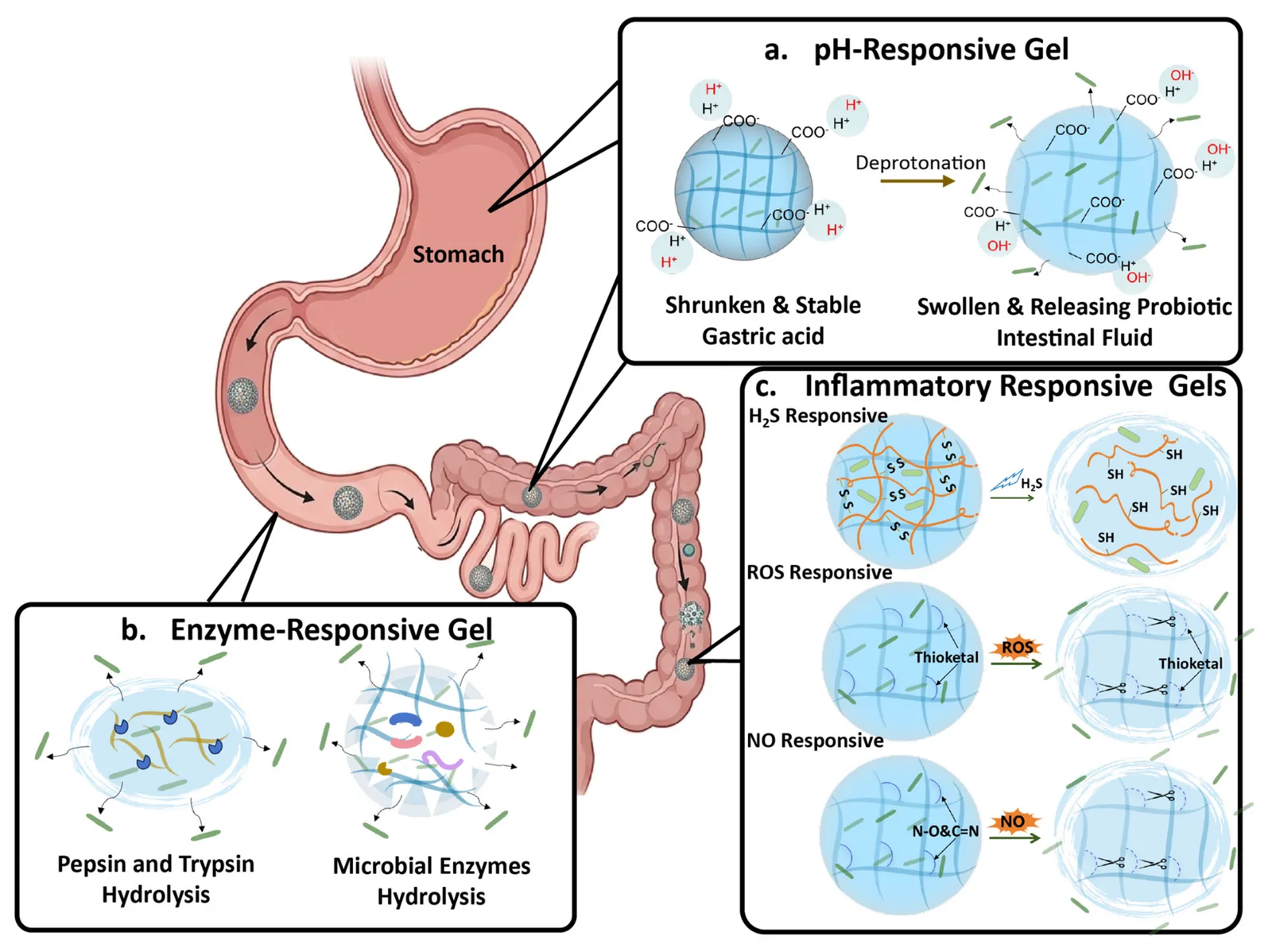
pH-Responsive gel, enzyme-responsive gel and inflammatory responsive gels.

High-pH-responsive frameworks are tailored specifically for intestinal transit, shielding probiotics from harsh stomach acids and triggering release in the neutral to weakly alkaline environment of the lower GI tract [75] (Figure 2). To guarantee bacterial viability in the highly acidic stomach, these encapsulation systems often incorporate acid-dissolvable components to actively consume hydrogen ions. A prime example is a self-adjusting gel-in-gel system, where probiotic-laden microgels are embedded within a protective gelatin and calcium carbonate macrogel. This outer matrix neutralizes acid to ensure high survival rates before undergoing temperature-responsive breakdown, subsequently releasing the mucoadhesive microgels for prolonged intestinal retention [79]. Beyond degradation, swelling-mediated platforms govern therapeutic release through the progressive, pH-dependent enlargement of the hydrogel’s interstitial pores. This multi-level responsiveness is typically achieved using natural and modified biopolymers that act as environmental switches. For instance, pH-sensitive polysaccharides contain acidic or basic moieties that accept or release protons based on the ambient pH. Acidic polysaccharides remain deprotonated and unswollen at low pH. However, as the environment becomes more alkaline, their ionization degree increases, promoting gel swelling. Basic polysaccharides exhibit the exact opposite response [80]. Similarly, the swelling behavior of proteins is dictated primarily by their isoelectric point [81]. When the environmental pH deviates from this point, proteins accumulate a net surface charge, creating electrostatic repulsion that forces the polymer network to expand and release its payload.

#### 3.1.2. Colonic Enzyme-Triggered Release

Enzyme-responsive delivery matrices leverage region-specific digestive activities to achieve the targeted release of encapsulated probiotics [82]. The gastrointestinal tract presents varying enzymatic environments, including pepsin in the stomach and trypsin in the intestines [83] (Figure 2). Researchers have engineered a delivery vehicle consisting of a Ca-alginate/protamine composite shell and a *Lactobacillus*-loaded Ca-alginate gel core. Because the protamine-integrated matrix resists gastric enzymes but is rapidly hydrolyzed by intestinal trypsin, this design significantly accelerates probiotic release in the small intestine [84]. Polysaccharide-based gels offer an effective mechanism for targeted delivery to the colon by resisting early digestive enzymes. Upon reaching the colon, microbial enzymes selectively cleave specific glycosidic linkages to facilitate cargo release [18].

Colonic reductases provide another vital trigger for polymeric carrier degradation and the localized activation of synergistic therapeutic agents. Enzymes such as azoreductase and nitroreductase are secreted by anaerobic bacteria resident in the colon. Coatings sensitive to azoreductase can enhance probiotic survival during transit and subsequently release compounds that reduce inflammation upon arrival in the colon [85]. This localized release suppresses inflammation and creates a favorable microenvironment for probiotic colonization to alleviate conditions including colitis [86]. Similarly, peptide hydrogels sensitive to nitroreductase maintain stability in early digestive stages but transition from a gel to a liquid state upon enzyme exposure [87]. This targeted transition enables precise probiotic release to effectively modulate the intestinal microbiota.

#### 3.1.3. Inflammation-Targeted Delivery Systems

Inflammation-responsive hydrogels designed for probiotic delivery represent a sophisticated convergence of materials science and gastrointestinal pharmacology, wherein carrier degradation and cargo liberation are orchestrated by specific pathophysiological signals (Figure 2). The therapeutic potential of free probiotics is severely constrained by poor gastrointestinal viability and a lack of spatial precision in conventional formulations. This necessitates advanced platforms that deliver probiotics through matrices exploiting the biochemical hallmarks of inflamed microenvironments, including elevated reactive oxygen species, nitric oxide, hydrogen sulfide, and glutathione, as endogenous triggers for localized release. By utilizing active tropism to enhance local accumulation and reduce systemic dissemination, these systems successfully navigate physiological barriers. For instance, inflammation-responsive hydrogels reacting to reactive oxygen species integrate cleavable moieties, such as boronic acid esters or thioketal linkages, within polymer backbones like alginate or hyaluronic acid. These structures hydrolyze exclusively in highly oxidative conditions, enabling targeted probiotic liberation through the gel matrix while concurrently scavenging excess radicals to mitigate tissue damage. In cutaneous applications, delivering *Lactobacillus rhamnosus* through inflammation-responsive hydrogels that react to both pH and reactive oxygen species can accelerate wound healing. This is achieved via synergistic antimicrobial and inflammation-mitigating actions without disrupting the native skin microbiome [88]. For gastrointestinal pathologies, the survivability of probiotics delivered through these gels can be further maximized. This involves fortifying the bacteria with antioxidant epigallocatechin gallate–manganese nanoparticles prior to encapsulation, thereby promoting robust colonic retention and the restoration of intestinal tight junction proteins [89].

Capitalizing on an orthogonal oxidative trigger, inflammation-responsive hydrogels reacting to nitric oxide leverage the elevated concentrations of this molecule characteristic of inflamed colonic tissues. Delivering probiotics through these platforms shields orally administered biotherapeutics from severe gastric degradation while initiating dissociation directly at inflammatory lesions. Polymeric gamma polyglutamic acid microcapsules engineered via microfluidics function as highly effective inflammation-responsive gels, providing robust cytoprotection by maintaining nearly 90% probiotic viability under simulated gastric conditions [90]. Upon exposure to environments rich in nitric oxide, rapid network collapse triggers probiotic release through the gel architecture to restore the intestinal mechanical barrier and microbial homeostasis. Expanding upon this approach, architectures responding to multiple stimuli have been utilized to deliver *Lactobacillus plantarum* through inflammation-responsive gels targeted to mucosal lesions in bacterial enteritis [91]. By concurrently releasing hydrogen sulfide and scavenging pathological nitric oxide, these matrices significantly enhance probiotic adhesion and attenuate mucosal inflammation during Salmonella infections.

In addition to oxidative stimuli, inflammation-responsive hydrogels are sensitive to reduction mechanisms. They exploit localized reducing agents, including endogenous glutathione or hydrogen sulfide produced by pathogens, to orchestrate localized probiotic delivery. These formulations incorporate reducible crosslinkers, notably disulfide or highly labile diselenide bonds, which undergo rapid cleavage via the exchange of thiols and disulfides in elevated redox states [92,93]. As a primary example, hyaluronic acid networks crosslinked by disulfide bonds act as inflammation-responsive gels that provide robust protection during gastrointestinal transit. These matrices subsequently degrade upon exposure to hydrogen sulfide derived from pathogens, allowing probiotics to be released through the gel network to enable competitive inhibition of pathogen adhesion and the restoration of intestinal eubiosis [94]. Ultimately, delivering probiotics through these dynamic inflammation-responsive hydrogels offers a highly controlled therapeutic alternative to traditional antibiotics by maximizing the localized efficacy of live biotherapeutics while circumventing antimicrobial resistance [95,96].

### 3.2. Mucoadhesive and Mucus-Penetrating Designs

**Figure 3:**
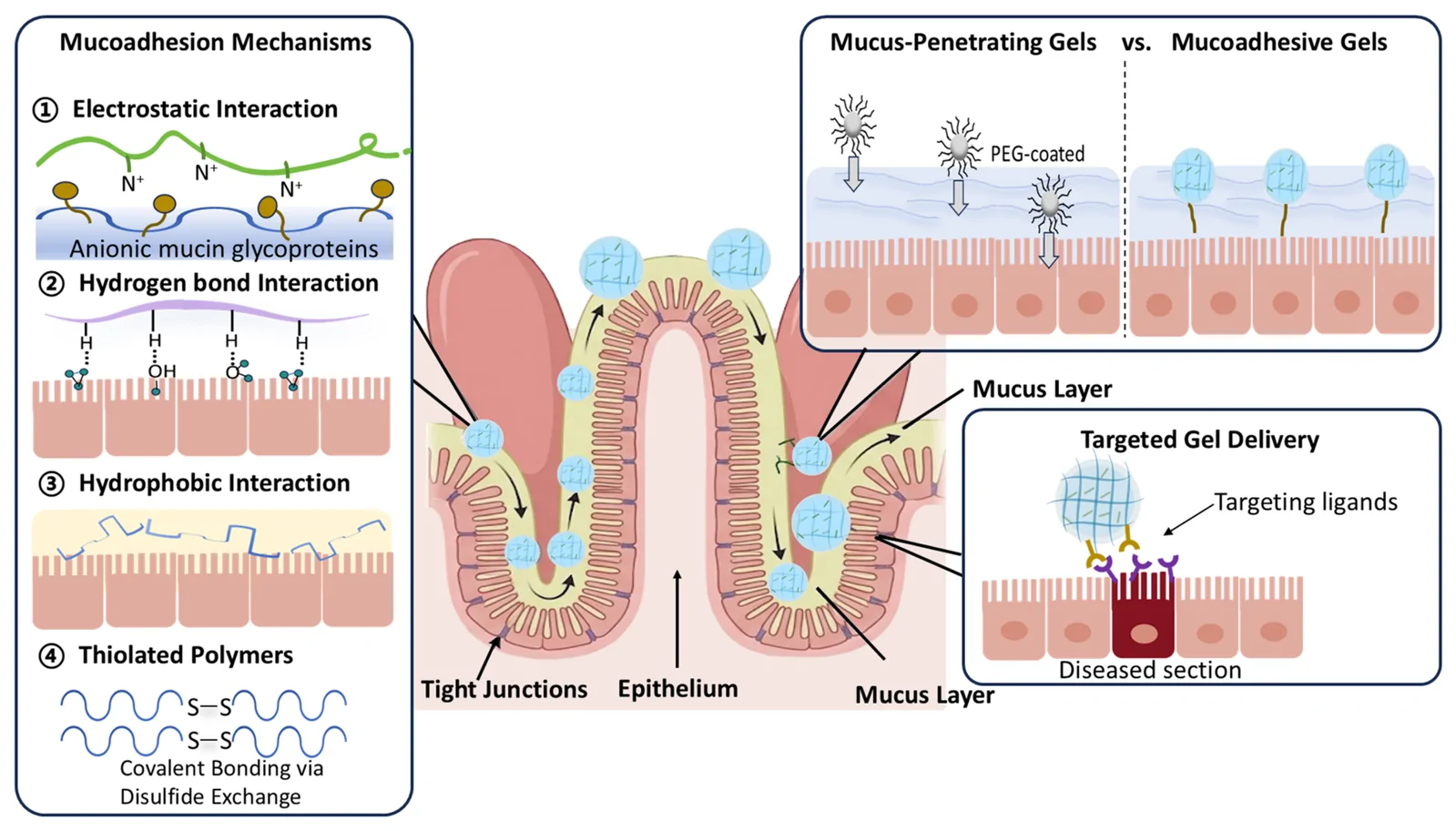
Mucoadhesive and Mucus-Penetrating Gel Designs.

To prolong gastrointestinal residence time and enhance probiotic colonization, gel delivery systems must navigate the dynamic mucosal barrier through mucoadhesive or mucus-penetrating mechanisms (Figure 3). Mucoadhesive gels anchor to the intestinal wall via electrostatic interactions and hydrogen bonding, as demonstrated by alginate and chitosan, or through robust covalent disulfide linkages formed between thiolated polymers and mucin glycoproteins [97,98]. Conversely, mucus-penetrating designs utilize densely packed and neutrally charged hydrophilic coatings, such as polyethylene glycol, to evade electrostatic entanglement and rapidly slip through the continuously shedding superficial mucus layer [99]. Advanced formulations increasingly integrate both strategies through dynamically responsive architectures. These systems utilize an outer penetrating corona to bypass superficial mucus clearance, followed by localized enzymatic or pH-triggered degradation that exposes a highly adhesive core, thereby ensuring deep tissue proximity and firm anchoring for optimal probiotic release [100].

Beyond merely achieving physical retention, overcoming colonization resistance requires advanced platforms that function as living hydrogels to orchestrate a coordinated and high-inoculum engraftment event [101]. Rather than acting as passive carriers, these dynamic bioreactors leverage the spatial confinement of the hydrogel matrix to allow entrapped probiotics to undergo sustained in situ proliferation, generating a metabolically active microenvironment prior to release [40,102]. This confinement prevents immediate competitive exclusion by indigenous microbiota and facilitates the localized accumulation of essential metabolites, including autoinducers and short-chain fatty acids. Upon mucosal adhesion, the hydrogel delivers a metabolically synchronized bacterial community, enabling the probiotics to collectively occupy mucosal binding sites and establish a stable niche before indigenous species can reassert dominance [103].

### 3.3. Advanced Colonization Platforms

Overcoming colonization resistance remains the main bottleneck in probiotic therapy [101]. Colonization requires administered microbes to adhere to the intestinal mucosa, survive environmental stressors, and establish a stably reproducing population. Traditional encapsulation mainly supports survival during gastrointestinal transit [104]. Recent work instead favors living hydrogels as dynamic bioreactors. These platforms create protected seeding depots at the mucosal interface. Encapsulated probiotics grow in situ. High cell densities within the matrix approach biofilm thresholds. Spatial confinement shields introduced strains from immediate competitive exclusion. It also concentrates essential metabolites. Autoinducers and short-chain fatty acids reinforce a sessile, adhesive phenotype [40].

In vivo studies show clear advantages for living delivery vehicles [102]. Adhering hydrogels release proliferating bacteria as synchronized units. This cohesive delivery lets probiotics occupy available binding sites before indigenous species respond. The living hydrogel acts as a functional bridgehead [103]. It coordinates high-inoculum events and lowers the barrier to stable intestinal engraftment. These features link mucosal retention, localized growth, and competitive occupancy. They support durable probiotic colonization.

## 4. Therapeutic Applications in Gastrointestinal and Systemic Health

### 4.1. Inflammatory Bowel Disease (IBD)

The pathogenesis of inflammatory bowel disease (IBD) is driven by mucosal barrier impairment, oxidative stress, and immune dysregulation [105]. To disrupt this cycle, hydrogels have emerged as multifunctional platforms capable of on-demand probiotic release and localized tissue modulation. Accordingly, these matrices are engineered to withstand gastrointestinal transit and selectively respond to inflammatory cues. For example, thiolated hyaluronic acid hydrogels exploit elevated intestinal hydrogen sulfide to trigger redox-responsive release exclusively at inflamed sites, while pH-sensitive sodium alginate microgels maintain gastric stability before undergoing intestinal degradation [106]. Additionally, fucoidan-based hydrogels utilize ultrafast self-healing properties to resist peristaltic shear, significantly enhancing probiotic viability across digestive fluids [107]. As a result, shielded and persistently engrafted within these matrices, the delivered probiotics exhibit robust metabolic activity and sustained survival. Mechanistically, this localized delivery synergizes with the hydrogel matrix: biomimetic fibrin-based hydrogels act as a “living bandage” to physically seal epithelial ulcers and promote localized tissue healing [108]. In a dextran sulfate sodium-induced colitis mouse model, probiotics released from keratin-based hydrogels have been shown to significantly upregulate critical tight junction proteins, thereby restoring mucosal barrier function [109]. Furthermore, the polymeric backbone often serves as a metabolic substrate. For example, inulin-based hydrogels undergo colonic fermentation. This process yields short-chain fatty acids, which provide an essential energy source for epithelial regeneration [110]. Concurrently, the sustained probiotic engraftment facilitated by these platforms downregulates pro-inflammatory cytokines, stabilizing the gut microbiota and suppressing overactivated adaptive immunity in DSS-induced colitis mice [111]. Although these findings from animal models highlight strong potential for mitigating IBD-associated inflammation, the intrinsic immunomodulatory properties of hydrogel polymers, particularly natural polysaccharides such as hyaluronic acid, further synergize with the delivered biotherapeutics to re-establish robust immune homeostasis within the inflamed mucosa, thereby warranting future clinical validation [112].

### 4.2. Colorectal Cancer (CRC)

The pathogenesis of colorectal cancer is intrinsically linked to gut microbiota dysbiosis. Plant-mediated synthesis of selenium nanoparticles has similarly demonstrated potent antimicrobial activity together with anticancer effects [113]. This positions hydrogel-delivered probiotics as a formidable adjunctive strategy with potential for oncological prevention and therapy [114]. However, the clinical efficacy of free probiotics is fundamentally hindered by extensive viability losses during gastrointestinal transit and suboptimal localized colonization. Hydrogel encapsulation effectively resolves these physiological bottlenecks. By shielding the bacterial payload from gastric acid and bile salts, hydrogels facilitate robust mucoadhesion and sustained, targeted release within the colonic microenvironment. Evidence from in vitro and animal studies suggests that probiotics exert multi-targeted anticarcinogenic effects [115]. Specifically, they competitively suppress pro-tumorigenic pathogens, downregulate carcinogenic enzymes, and produce bioactive metabolites like butyrate to induce colonocyte apoptosis. To address this pathological condition and its associated tumor microenvironment barriers, these living therapeutics attenuate chronic mucosal inflammation and fortify the intestinal epithelial barrier. This results in markedly superior tumor-suppressive efficacy in preclinical murine models compared to unencapsulated formulations [116]. Transitioning these advanced hydrogel delivery platforms into clinical oncology mandates rigorous scrutiny of their long-term safety and translational viability. A primary imperative is evaluating the biocompatibility, potential systemic absorption, and prolonged accumulation of polymeric matrix materials within the structurally compromised intestinal architecture of oncological patients [117]. The robust therapeutic outcomes observed in established preclinical systems must be validated through large-scale human cohorts. To fully harness the clinical utility of gut-targeted living therapeutics, future research must pivot toward personalized formulations guided by comprehensive host microbiome profiling to ensure optimal strain engraftment. Ultimately, integrating hydrogel-encapsulated probiotics with conventional oncological paradigms, particularly as synergistic adjuvants to immune checkpoint inhibitors, represents a critical frontier in amplifying systemic anti-tumor immunity [118], though its actual clinical benefit in cancer patients remains to be established.

### 4.3. Gut–Brain Axis and Other Disorders

Beyond localized intestinal pathologies, the therapeutic horizon for probiotic delivery has expanded to encompass systemic conditions, particularly those governed by the microbiota–gut–brain axis [119]. This bidirectional communication network is highly responsive to neuroactive molecules and short-chain fatty acids synthesized by specific probiotic strains. However, the clinical efficacy of unencapsulated psychobiotics is frequently compromised by rapid luminal clearance, which prematurely terminates the continuous metabolite production required for distal neural signaling. Hydrogel encapsulation effectively circumvents these pharmacokinetic limitations by establishing engineered living hydrogels that function as persistent, metabolically active microbial depots within the gastrointestinal tract [120]. Consequently, protected and anchored within these matrices, the delivered probiotics exhibit sustained viability and continuous metabolic output. Beyond the gut–brain axis, the utility of this continuous biochemical stimulation extends well beyond neurological applications, offering robust interventions for a broader spectrum of systemic and metabolic disorders. In conditions characterized by pervasive microbial dysbiosis, such as obesity and metabolic syndrome, the sustained regulation of the gut microenvironment is paramount. Recent advancements demonstrate that specialized carriers, including beta-glucan superabsorbent hydrogels, can effectively reverse obesity-associated metabolic dysfunction in experimental models by delaying gastric emptying and fortifying intestinal barrier integrity [121]. Similarly, microencapsulated strains have proven significantly more effective than free bacterial suspensions in ameliorating distal organ damage, such as hepatorenal injury. Ultimately, by transforming vulnerable probiotic formulations into stable, long-acting living therapeutics, advanced hydrogel platforms successfully bridge the mechanistic gap between localized colonic engraftment and the therapeutic biochemical modulation of distal organs.

## 5. Current Challenges, Future Perspectives, and Conclusions

In conclusion, hydrogel-based delivery systems represent a transformative paradigm in the management of severe intestinal disorders, evolving probiotic administration into a multi-stage “protection–targeting–release–synergy” therapeutic modality. By shielding sensitive microorganisms within functional matrices, these biomaterials successfully overcome the fundamental limitations of poor oral bioavailability and diminished viability during gastrointestinal transit. Operating as localized bioreactors, hydrogels work synergistically with live biotherapeutics to execute multi-target interventions. This integrated approach effectively restores gut homeostasis by directly antagonizing pathogens, reinforcing the epithelial barrier, and modulating mucosal immunity from pro-inflammatory to anti-inflammatory states.

It is essential to distinguish protective encapsulation from functional colonization. Unlike conventional hydrogels that act as passive physical containers, engineered living hydrogels are defined by an active cellular–matrix symbiosis, where encapsulated cells maintain continuous metabolic activity and dynamic interactions within the polymer network. While hydrogel encapsulation frequently enhances probiotic viability during processing, storage, and gastrointestinal transit, true living systems demand sustained in situ viability and metabolic output to drive therapeutic functions. Improved survival does not necessarily signify engraftment, sustained metabolic activity, or durable in vivo microbiota modulation. Most of the current evidence supports enhanced delivery and survival, whereas direct evidence for colonization and functional health outcomes remains scarce and is often preclinical or correlational. Future studies should therefore quantify and report colonization persistence, spatial localization, metabolic activity, and microbiota-level alterations together with clinically relevant endpoints, and should explicitly acknowledge where causal relationships remain unestablished.

Despite this unprecedented therapeutic potential, the clinical translation of these “living materials” remains hindered by complex regulatory frameworks, stringent safety evaluation requirements, and industrial bottlenecks such as manufacturing scalability, batch-to-batch reproducibility, cross-linking uniformity, residual solvent and cross-linker levels, drying methods, sterile manufacturing considerations for live probiotics, encapsulation-induced cellular stress, and long-term shelf-stability. Living hydrogels face additional challenges beyond those of conventional encapsulation, especially nutrient supply and diffusion within the matrix. Poor diffusion reduces metabolic activity. Metabolic activity must also be maintained during storage and after administration, as encapsulated cells need to remain active rather than merely survive. These challenges should be addressed together with biosafety, reproducibility, and regulatory issues. Safety, residual materials, and regulatory positioning also require systematic attention. Chemical cross-linkers, residual metal ions, and the long-term fate of non-digestible matrix components should be evaluated for toxicity, accumulation, and clearance. Regulatory classification will depend on intended use and primary mode of action: food- or nutrition-oriented hydrogels may be regulated as food additives, novel foods, or generally recognized as safe substances, whereas therapeutic hydrogels may be classified as medical devices, drug-delivery systems, or combination products in major jurisdictions such as the U.S. Food and Drug Administration, the European Medicines Agency, the European Food Safety Authority, and the National Medical Products Administration. Early regulatory consultation and systematic safety assessment are therefore essential for clinical and commercial translation. Because most reported systems remain at the laboratory scale, realistic pathways to food- or pharmaceutical-grade probiotic hydrogel production will require reproducible process design, robust quality control, pilot-scale validation, and early consideration of regulatory requirements.

Overcoming these hurdles necessitates a strategic pivot. Precision-engineered, active platforms should be developed and tailored to distinct pathological microenvironments. Future research must address strain-specific physiological requirements, advance dose standardization, and overcome translational limitations. Findings from simulated gastrointestinal conditions and animal models should not be directly extrapolated to human cohorts. Meanwhile, precise mechanistic interactions within the hydrogel–probiotic–host triad must be elucidated, and outcomes must be validated in large-scale human studies. Next-generation living hydrogels may improve the management of IBD and related gastrointestinal disorders, but human evidence is limited and translational barriers remain.

## Data Availability

The original contributions presented in this study are included in the article. Further inquiries can be directed to the corresponding author.
